# In Vitro Assessment of the Genotoxic Potential of Pristine Graphene Platelets

**DOI:** 10.3390/nano11092210

**Published:** 2021-08-27

**Authors:** Andrea Malkova, Tereza Svadlakova, Avni Singh, Martina Kolackova, Radka Vankova, Pavel Borsky, Drahomira Holmannova, Adam Karas, Lenka Borska, Zdenek Fiala

**Affiliations:** 1 Institute of Preventive Medicine, Faculty of Medicine in Hradec Kralove, Charles University, 50003 Hradec Kralove, Czech Republic; svadlakovat@lfhk.cuni.cz (T.S.); BORSKYP@lfhk.cuni.cz (P.B.); holmd9ar@lfhk.cuni.cz (D.H.); karasad@lfhk.cuni.cz (A.K.); borka@lfhk.cuni.cz (L.B.); fiala@lfhk.cuni.cz (Z.F.); 2 Institute of Pathological Physiology, Faculty of Medicine in Hradec Kralove, Charles University, 50003 Hradec Kralove, Czech Republic; 3 Institute of Clinical Immunology and Allergology, University Hospital Hradec Kralove and Faculty of Medicine in Hradec Kralove, Charles University, 50005 Hradec Kralove, Czech Republic; singhav@lfhk.cuni.cz (A.S.); kolackovam@lfhk.cuni.cz (M.K.); vankovr@lfhk.cuni.cz (R.V.)

**Keywords:** pristine graphene platelets, THP-1, genotoxicity, cytotoxicity, oxidative stress, immunotoxicity, micronucleus test

## Abstract

(1) Background: Graphene is a two-dimensional atomic structure with a wide range of uses, including for biomedical applications. However, knowledge of its hazards is still limited. This work brings new cytotoxic, cytostatic, genotoxic and immunotoxic data concerning the in vitro exposure of human cell line to two types of graphene platelets (GP). It also contributes to the formation of general conclusions about the health risks of GP exposure. (2) Methods: In vitro exposure of a THP-1 cell line to three concentrations of two GP over 40 h. The cytotoxic potential was assessed by the measurement of LDH and glutathione (ROS) and by a trypan blue exclusion assay (TBEA); the cytostatic and genotoxic potential were assessed by the cytokinesis-block micronucleus (CBMN) test; and the immunotoxic potential was assessed by the measurement of IL-6, IL-10 and TNF-α. (3) Results: We found a significant dose-dependent increase in DNA damage (CBMN). The lowest observed genotoxic effect levels (LOGEL) were 5 µg/mL (GP1) and 30 µg/mL (GP2). We found no significant leaking of LDH from cells, increase in dead cells (TBEA), induction of ROS, increased levels of cytostasis, or changes in IL-6, IL-10 and TNF-α levels. (4) Conclusions: The genotoxicity increased during the short-term in vitro exposure of THP-1 to two GP. No increase in cytotoxicity, immunotoxicity, or cytostasis was observed.

## 1. Introduction

Graphene is a two-dimensional (2D) atomic structure with a honeycomb lattice that has extraordinary properties, such as elasticity, mechanical stiffness and strength, high thermal and electrical conductivity, high transparency, a large specific surface area and high molecular adsorption [1,2,3]. The biological applications of graphene include antibacterial and antiviral applications, cancer targeting and therapy, drug delivery, photothermic therapy, tissue engineering, DNA sequencing, stem cell technology and probes for fluorescent tracking [3].

Despite the great potential of graphene to have a wide application in biomedicine, it is still not clearly elucidated whether it is fully biocompatible [4]. The drawing of general conclusions about the degree of health hazard in human exposure to graphene is very complicated because the interactions of graphene with an organism are influenced by many physicochemical properties, such as the size, shape, purity, number of layers, surface charge, hydrophilicity, synthesis methods, dispersion state, oxidative state and the route and dose of administration [2,3,5].

The blood components circulate throughout the body and are in contact with tissue cells, organ cells and xenobiotics. From this point of view, the blood cells represent a suitable model for the evaluation of interactions between xenobiotics and tissue/organ cells and the related assessment of health hazards [1]. Leukocytes represent the front line of immune defense against pathogens or foreign materials and are responsible for the elimination of exogenous and endogenous materials [1]. Leukocytes release many cytokines, which modulate the function and expression of cell surface markers and chemokines [6]. The human acute monocytic leukemia cell line (THP-1 cell line) is considered as a suitable model for in vitro toxicological/biocompatibility studies [4].

The toxic potential of graphene-based materials (G-BNMs) has so far been evaluated mainly for graphene oxide (GO) [4,7,8,9,10,11,12,13], but the data obtained on pristine graphene and human cell lines are limited.

To our knowledge, this is the first study assessing the cytotoxic, cytostatic, genotoxic and immunotoxic potential of non-activated (suspension) THP-1 cells exposed in vitro to two types of pristine graphene platelets (GP).

The aim of the presented article is to bring new cytotoxic, cytostatic, genotoxic and immunotoxic data concerning the consequences of in vitro exposure of the human THP-1 cell line to two types of GP. The work thus contributes to the formation of general conclusions about the degree of risk of GP exposure.

## 2. Materials and Methods

### 2.1. Graphene Platelets

Two randomly selected representative types of pristine GP were used in the study. GP1 were purchased from PlasmaChem GmbH (Berlin, Germany, product number PL-P-G750) as a powder and GP2 were kindly donated by CRANN (the Center for Research on Adaptive Nanostructures and Nanodevices, Trinity College, Dublin, Ireland) as a powder.

Detailed physicochemical characterization and a full description of the preparation of stock suspensions of GP (250 µg/mL) were stated in our recent work [14]. The basic characterization is briefly summarized in Table 1. The thickness of both GP was up to 4 nm.

#### Contamination of Graphene Platelets (Endotoxin Content)

The presence of a biologically active lipopolysaccharide (LPS) in GP stock solutions was evaluated using the cell-based assay HEK-Blue™ LPS Detection Kit 2 (InvivoGen, San Diego, CA, USA), following the manufacturer’s protocol. HEK-Blue™-4 cells used in this assay were cultivated in Dulbecco’s modified Eagle’s High Glucose medium without phenol red (DMEM; Corning, New York, NY, USA) with the addition of 10% heat-inactivated ultra-low endotoxin fetal bovine serum (FBS_LE_; Biosera, France), 2 mM L-alanyl-L-glutamine (GlutaMAX; Life Technologies, Carlsbad, CA, USA), Normocin (100 µg/mL; InvivoGen, San Diego, CA, USA) and the selection antibiotics 250X HEK-Blue™ Selection (InvivoGen, San Diego, CA, USA). The working concentrations of GP were tested, along with the spiked samples, with LPS (0.1 EU/mL) for the evaluation of possible interferences. The results were calculated according to the concentration grade of the standard endotoxin from the *Escherichia coli* serotype 055:B5 (0.01–1 EU/mL).

### 2.2. Exposure of Human THP-1 Cell Line to GP

The human THP-1 cell line (human acute monocytic leukemia cell line) was purchased from the European Collection of Authenticated Cell Cultures (ECACC, Salisbury, UK). Cells were incubated in a humidified atmosphere of 5% CO_2_ at 37 °C and cultivated in RPMI 1640 medium without phenol red (Corning, New York, NY, USA), with the addition of 10% heat-inactivated FBS_LE_, 2 mM GlutaMAX, 1 mM sodium pyruvate (Life Technologies, Carlsbad, CA, USA), 10 mM HEPES, 0.05 mM 2-mercaptoethanol, as well as with penicillin (100 U/mL) and streptomycin (100 µg/mL) (Sigma-Aldrich, St. Louis, MI, USA). For experiments, cells were used as suspension cells without activation. In all exposure experiments, cells with sodium cholate (used as a solvent for GP suspension preparation in 0.0048% concentration, corresponding to the concentration in 60 µg/mL), water (corresponding to the concentration in 60 µg/mL) and untreated cells were used as controls. The exposure of THP-1 cells to both types of GP and controls took 40 h in all experiments because 40 h corresponds to the 1.5-cell cycle length. The concentrations of GP used in all experiments were low (5 µg/mL), medium (30 µg/mL) and high (60 µg/mL).

### 2.3. Cell Viability/Cytotoxicity

#### 2.3.1. WST-1

The colorimetric assay WST-1 was used for measuring the viability of exposed THP-1 cells. The cells were seeded in 96-well plates at a concentration of 2 × 10^5^ cells/mL, and exposed to three different concentrations of GP over a period of 40 h. In the WST-1 assay, 10 µL of WST-1 solution (Cell Proliferation Reagent WST-1, Roche, Basel, Switzerland) was mixed with 100 µL of cell suspension and incubated for 3 h. For the evaluation of possible interferences, previously untreated cells were incubated with WST-1 reagent, together with additional GP for 3 h. Suspensions were further centrifuged for 10,000× *g* for 10 min to wash out the GP and transferred to a new flat-bottomed 96-well plate. Absorbance was measured in a microplate spectrophotometer, Synergy HTX (Biotek, Winooski, VT, USA), at 440 nm, with 690 nm set as the reference wavelength.

#### 2.3.2. Cell Membrane Integrity

The impact of two types of GP on the cell membrane integrity of the THP-1 cell line was assessed by the measurement of lactate dehydrogenase (LDH) (CyQUANT™ LDH Cytotoxicity Assay, Invitrogen, Carlsbad, CA, USA) leakage after 40 h of exposure of the THP-1 cells (2 × 10^5^ cells/mL). LDH is a cytosolic enzyme that is released after damage to the plasma membrane in the cell culture medium and serves as a reliable and well-established marker of cell membrane damage and cytotoxicity [5,14,15]. The cells’ supernatants were centrifuged for 10,000× *g* for 10 min to wash out the GP and transferred to a new 96-well plate with a flat bottom. The LDH assay was performed according to the manufacturer’s protocol. Absorbance was measured in a microplate spectrophotometer, Synergy HTX (Biotek, Winooski, VT, USA), at 490 nm, with 690 nm set as the reference wavelength.

Membrane integrity and the cell number of THP-1 cells after 40 h of exposure to the highest concentration of two types of GP (60 µg/mL) were also assessed with a trypan blue exclusion assay (TBEA). During the trypan blue (TB) exclusion assay, the live cells with intact cytoplasmic membranes are not stained by the TB dye, whereas the dead cells are stained. The dye penetrates into the cells due to changes in the integrity of the cytoplasmic membrane [16]. THP-1 cells exposed to GP and controls were harvested and resuspended in fresh RPMI medium. Next, 10 µL of cell suspension was added to 10 µL of 0.4% solution of TB (Gibco, Thermo Fisher Scientific, Carlsbad, CA, USA) and mixed. A Bürker chamber was used for cell counting. To test whether the presence of GP could affect cell viability in co-exposure with cytochalasin B (PanReac AppliChem, Darmstadt, Germany), used for a further experiment, the cells were stained after another 30-h incubation. Only the unstained (live) cells were calculated for an evaluation of the total number of cells after exposure to GP, using an inversion microscope, Eclipse Ts2 (Nikon, Minato, Japan), with a 10× objective lens (total magnification 100×).

### 2.4. Oxidative Stress

To assess the possible oxidative stress of treated THP-1 cells, the glutathione (GSH) concentration in cell lysates was determined using a glutathione colorimetric detection kit (Invitrogen, Thermo Fisher Scientific, Carlsbad, CA, USA). After 40 h of exposure to both types of GP, cells were collected and processed according to the manufacturer’s protocol. Absorbance was measured in a microplate spectrophotometer Synergy HTX (Biotek, Winooski, VT, USA) at 405 nm. To determine the oxidized glutathione (GSSG), lysates were treated with 2-vinylpyridine (2PVP; Sigma-Aldrich, St. Luis, MO, USA) for 1 h in RT. The concentrations of total GSH and GSSG were determined according to a standard curve for GSH and GSSG, respectively. The concentration of free GSH was calculated by subtracting the GSSG concentration values from the total GSH.

### 2.5. Cytokinesis Block Micronucleus Test

A Cytokinesis Block Micronucleus (CBMN) test was performed according to a modified method described by Fenech [17] and the OECD [18]. Briefly, 2.5 mL of THP-1 cells, in a concentration of 2 × 10^5^ cells/mL, were seeded in 6-well plates with exposure to the two types of GP, to 5 and 20 ng/mL of cytosine arabinoside (Sigma-Aldrich, St. Louis, MO, USA; positive controls), and to all controls described above for 40 h at 37 °C and 5% CO_2_. After 40 h of incubation, positive cultures were washed twice in PBS and the medium was replaced with a fresh one. Into all cultures, we added cytochalasin B (PanReac AppliChem, Darmstadt, Germany) in a final concentration of 5.0 µg/mL. After another 30 h of cultivation in the same conditions, the cells were collected by centrifugation (200× *g*, 8 min), and were fixed in the first step by using 8.0 mL of methanol:acetic acid (3:1) with 225 µL of 36–38% formaldehyde (Penta, Prague, Czech Republic), and in the second and third steps by using 8.0 mL of methanol: acetic acid (3:1) for each sample. After fixation, the cells were dropped onto humid chilled slides and were left to dry overnight at RT. The slides were stained with 5% Giemsa-Romanowski solution (Penta, Prague, Czech Republic) for 10 min on the next day. The analysis was performed with a 40× objective lens (total magnification 400×) using a B-383PLi microscope (Optika, Ponteranica, Italy). The number of cells with micronuclei (MN), nuclear buds (NBUDs) and nucleoplasmic bridges (NPBs) per 1000 binucleated cells (*BNC*) was scored in each sample. The cytokinesis-block proliferation index (*CBPI*), replication index (*RI*) and nuclear division index (*NDI*) were calculated by counting a minimum of 500 cells for each sample, including mononuclear (*MONOC*), binucleated (*BNC*), trinucleated (*TRINC*) and tetranucleated (*TETRC*) cells, according to Equations (1)–(3), respectively:

(1) $$CBPI=\frac{nMONOC+2\times nBNC+3\times \left(nTRINC+nTETRC\right)}{\left(total\;number\;of\;cells\right)}$$

(2) $$RI=\frac{\left(\frac{nBNC+2\times \left(nTRINC+nTETRC\right)}{\left(total\;number\;of\;cells\right)}\right)T}{\left(\frac{nBNC+2\times \left(nTRINC+nTETRC\right)}{\left(total\;number\;of\;cells\right)}\right)C}$$

where *T* is the tested cell culture, and *C* is the control culture.

(3) $$NDI=\;\frac{\left(nMONOC+2\times nBNC+3\times nTRINC+4\times nTETRC\right)}{\left(total\;number\;of\;cells\right)}$$

The percentage (%) of cytostasis was calculated according to Equation (4), where *T* is the tested cell culture, and *C* is the control culture:

(4) $$\%\;cytostasis=100-100\times \frac{\left(CBPI\right)T}{\left(CBPI\right)C}$$

All exposed cell cultures were used in duplicates, with cholate and water in triplicate and control cell cultures in tetraplicate, and the experiments were repeated three times. Therefore, for MN, NBUDs and NPBs assessment, it was scored as 6000 *BNC* for every tested concentration of GP and cytosine arabinoside, 9000 *BNC* for cholate and water, and 12,000 *BNC* for the negative control in total.

### 2.6. Cytokine Secretion

IL-6 and IL-10 in supernatants of THP-1, exposed to GP for 40 h, were detected by the cell-based assays using the human reporter cell line HEK-Blue™ IL-6 cells and HEK-Blue™ IL-10 cells (Invivogen, San Diego, CA, USA), respectively. Both cell lines were maintained in DMEM, supplemented with 10% FBS_LE_, 2 mM GlutaMAX, Normocin and the selection antibiotics, 250X HEK-Blue™ Selection. HEK-Blue™ cells responded specifically to IL-6/IL-10. The specific detection of bioactive cytokine, via a colorimetric assay of enzyme activity of the expressed reporter gene SEAP, is achieved by binding the specific IL to its receptor on the surface of HEK-Blue™. SEAP was quantified using QUANTI-Blue™ (Invivogen, San Diego, CA, USA), a SEAP detection medium that turns blue in its presence. Absorbance was measured in a microplate spectrophotometer, Synergy HTX (Biotek, Winooski, VT, USA), at 630 nm wavelength. The levels of TNF-α in supernatants of THP-1 exposed to GP for 40 h was detected by using a Human TNF-α Quantikine ELISA Kit (R&D Systems, Minneapolis, MN, USA), according to the manufacturer’s protocol.

### 2.7. Statistical Analysis

Unless stated otherwise, the data are shown as mean values (*n*-tests = 3) ± standard deviation and are normalized to the control. Changes have been considered significant for *p*-values < 0.05. Based on the Shapiro–Wilk test of normality, either the parametric or nonparametric analysis of variance (ANOVA), followed by either Dunnett’s test or the Kruskal–Wallis test, were performed using GraphPad Prism™ software, version 8.2.1 329 (GraphPad Software Inc., San Diego, CA, USA).

## 3. Results

### 3.1. Cell Viability/Cytotoxicity

Due to the detected interference of GP1 with a WST-1 assay, we excluded this test from the viability assessment (Figure S1, supplementary data).

**Figure 1:**
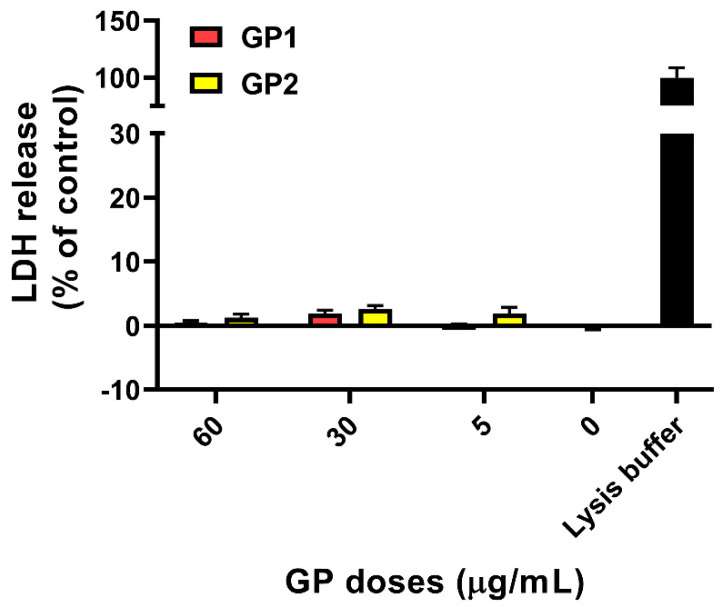
LDH release from THP-1 cells after 40 h of exposure to GP. Data are reported as mean ± standard deviation. LDH release (%) was calculated according to the absorbances of untreated cells (0 µg/mL) and positive control (lysis buffer).

**Figure 2:**
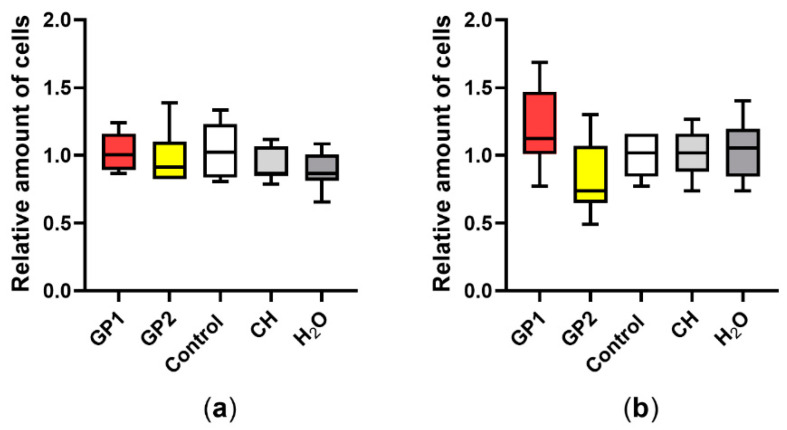
Total cell number according to the trypan blue exclusion assay: (**a**) comparison of unexposed THP-1 (Control), THP-1 exposed to GP, sodium cholate (CH) and H_2_O after 40 h of exposure; (**b**) after cotreatment with cytochalasin B (5 µg/mL). Data are normalized to the control (untreated THP-1) and are presented in a boxplot with a median with an interquartile range; *n* = 9.

According to our data (Figure 1), there was no significant release of LDH after 40 h of exposure of THP-1 to GP1 and GP2. The findings were confirmed by a trypan blue exclusion assay, where we did not observe any increase in the number of damaged cells. Furthermore, there was no significant change between the total number of cells exposed to GP when compared to controls after 40 h of exposure (Figure 2a). The presence of GP also had no effect on cell viability after the addition of cytochalasin B and a 70-h incubation period (Figure 2b).

### 3.2. ROS Generation

**Figure 3:**
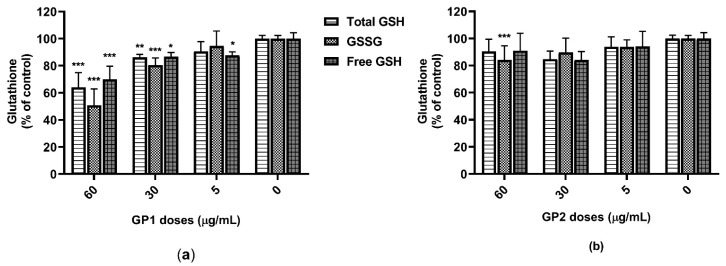
Glutathione content in THP-1 lysates after 40 h of exposure to GP: (**a**) content of total glutathione (GSH), oxidized glutathione (GSSG) and free GSH after exposure to GP1; (**b**) content of total GSH, GSSG and free GSH after exposure to GP2. Data are presented as a percentage (%) of untreated control (0 µg/mL) and are visualized as mean ± standard deviation. * *p*-value < 0.05; ** *p*-value < 0.01; *** *p*-value < 0.001.

Results from the GSH assay have shown a dose-dependent decrease of all the total GSH, GSSG and free GSH in THP-1 cells that were exposed to GP1 (Figure 3a). In contrast, 40 h of incubation with GP2 led to a significant decrease in GSSG only at the highest dose tested (60 µg/mL; Figure 3b). We did not prove any possible interference between the GSH assay and GP.

### 3.3. Cytostasis

**Figure 4:**
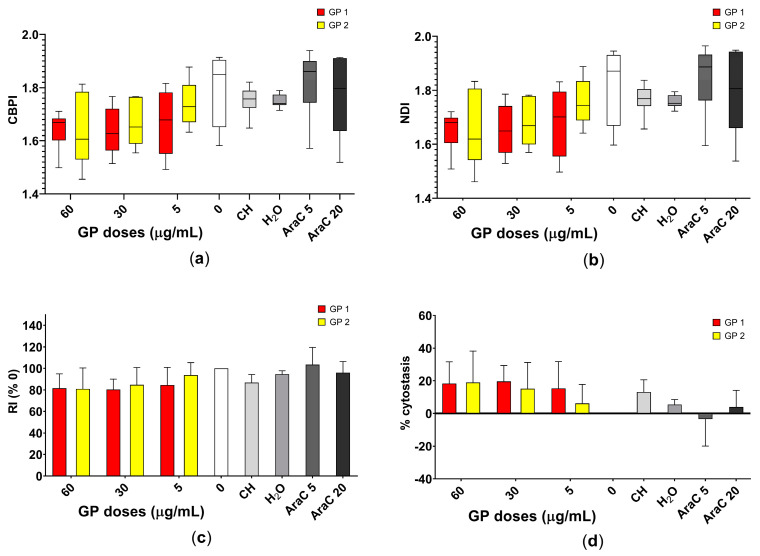
Assessment of the cell proliferation of THP-1 cells after 40 h of exposure to two types of graphene platelets (GP1, GP2) in a concentration of 0–60 µg/mL by CBMN. Assessment of the (**a**) cytokinesis-block proliferation index (*CBPI*); (**b**) nuclear division index (*NDI*); (**c**) replication index (*RI*) and (**d**) approximation of the percentage of cytostasis. Data are presented in a boxplot with a median with interquartile range or as mean ± standard deviation. CH—sodium cholate (in a concentration corresponding to 60 µg/mL); H_2_O—water (in a concentration corresponding to 60 µg/mL); AraC 5 or 20—cytosine arabinoside (in concentrations of 5 and 20 ng/mL).

In the study, the dose-dependent decrease in *CBPI*, *NDI*, *RI* and their associated percentages of cytostasis, after 40 h of exposure of the suspension THP-1 cells to GP1 and GP2, was noted as described in Figure 4. Both types of GP showed a potential to decrease the proliferation potential of THP-1 cells, but this decrease was not statistically significant.

### 3.4. Genotoxicity

**Figure 5:**
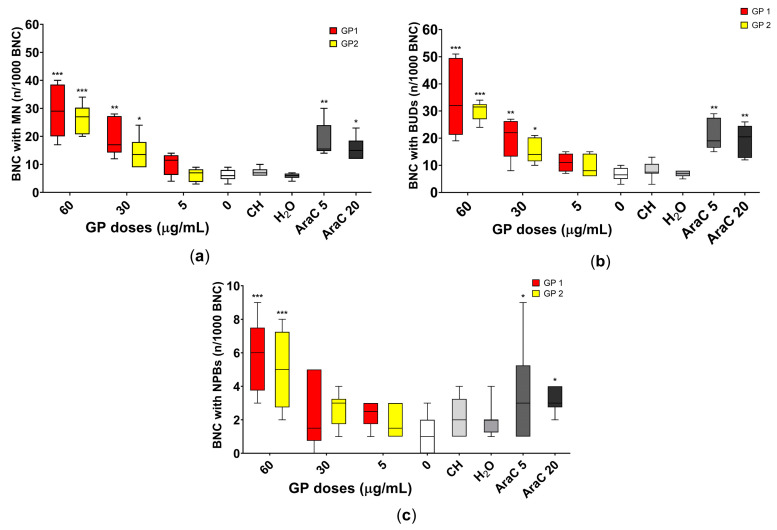
Assessment of the DNA damage of THP-1 cells after 40 h of exposure to two types of graphene platelets (GP1, GP2) in a concentration of 0–60 µg/mL by CBMN. Assessment of (**a**) number of binucleated cells (*BNC*) with micronuclei (MN); (**b**) *BNC* with nuclear buds (BUDs); (**c**) *BNC* with nucleoplasmic bridges (NPBs). Data are presented in a boxplot with a median in the interquartile range. CH—sodium cholate (in a concentration corresponding to 60 µg/mL); H_2_O—water (in a concentration corresponding to 60 µg/mL); AraC 5 or 20—cytosine arabinoside (in concentrations of 5 and 20 ng/mL); * *p*-value < 0.05; ** *p*-value < 0.01; *** *p*-value < 0.001.

**Figure 6:**
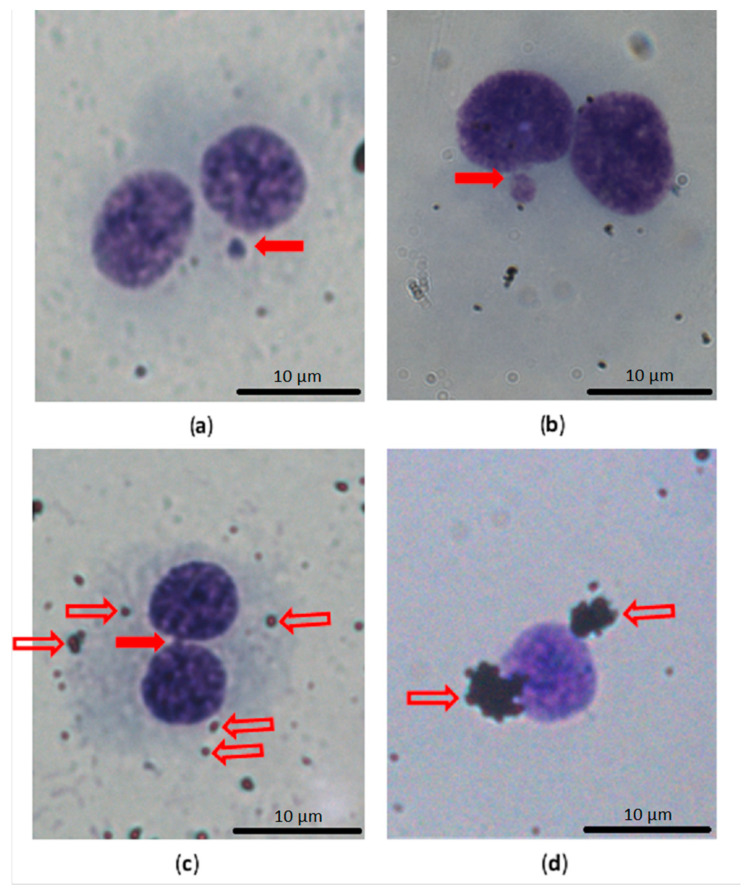
Example of the main assessed findings in CBMN test, with a comparison of the nucleus with cytoplasm and naked nucleus and GP clusters. (**a**) Binucleated cell (*BNC*) with one micronucleus (solid arrow); (**b**) *BNC* with a nuclear bud connected with the main nucleus by a narrow bridge (solid arrow); (**c**) *BNC* with a nucleoplasmic bridge (solid arrow) and small agglomerates of GP in the cytoplasm (on the cell surface; outline arrows); (**d**) naked nuclei without cytoplasm in the CBMN test were more often surrounded by clusters of GP (outline arrows).

The dose-dependent increase in DNA damage, mainly in the number of MN, NBUDs and NPBs in 1000 *BNC*, was noted as shown in Figure 5, in THP-1 cells in suspension after 40 h of exposure to both GPs. Representative pictures of the assessed parameters are shown in Figure 6.

### 3.5. Inflammatory Response

**Figure 7:**
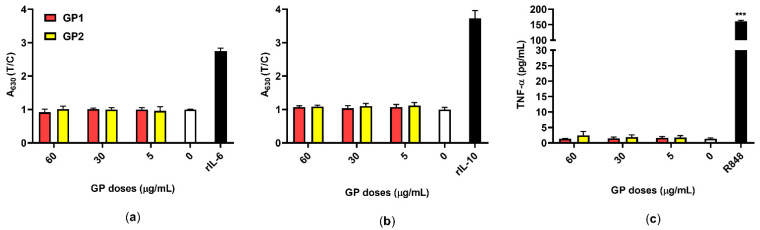
Inflammatory responses of THP-1 in response to GP (T; 5–60 µg/mL) compared to untreated control (0 µg/mL). (**a**) The release of IL-6 was measured using the reporter HEK-Blue™ IL-6 cells ELISA, rIL-6 (3 ng/mL) was used as an assay control; (**b**) the release of IL-10 was measured using the reporter HEK-Blue™ IL-10 cells, rIL-10 (10 ng/mL) was used as an assay control. Data are normalized to the control (0 µg/mL) and are presented as mean ± standard deviation; (**c**) the release of TNF-α was measured using ELISA, and R848 (10 µM) served as a positive control. Data are presented as mean ± standard deviation. *** *p*-value < 0.001.

The secretion of the pro-inflammatory cytokines IL-6 and TNF-α, as well as that of the anti-inflammatory cytokine IL-10, were quantified after 40 h of exposure of the THP-1 to both GP. Neither GP1 nor GP2 caused the release of these cytokines into the supernatants (Figure 7).

## 4. Discussion

### 4.1. Cytotoxicity of GP

#### 4.1.1. Interference between GP and Cytotoxicity Assays

We found interferences in the form of the decreasing activity of WST-1 in the presence of GP1 (Figure S1b). The same effect was also observed in the MTT assay in our previous study (data not shown). This false-positive increase of cytotoxicity is usually attributed to optical interferences between tetrazolium salts and nanomaterials (GP), the adsorbing of tetrazolium salt onto a nanomaterial surface, or the transfer of electrons [19]. Regarding the existence of these interferences, it was complicated to decide whether the decrease in WST-1 activity after exposure to GP1 was as a result of affecting cell metabolism or the result of interferences. Therefore, we decided to exclude the WST-1 assay results from our evaluation of GP cytotoxicity.

We did not observe any interferences in the case of LDH or TBEA. A modified LDH assay is suitable for testing the cytotoxicity of carbon-based NMs [20]. As for TBEA, it is highly recommended that researchers count the cells under the microscope by hand, as any NM aggregates that are present may “mimic” cells in an automated cell counter [21].

#### 4.1.2. Cytotoxic Potential of GP

Graphene-based nanomaterials (G-BNMs) have broad physicochemical features and toxicological profiles. It was recognized that the cellular uptake of GP can be influenced by the particles’ shape, size, and morphology, whereas their interaction with proteins, micronutrients, and biomolecules can be affected by the GP functional groups [2]. Thus, it is very complicated to draw any general conclusions about the cytotoxicity of G-BNMs [22].

Concerning the impact of GP on membrane integrity, some sources in the literature report the direct mechanical penetration of few-layer graphene platelets [23] or graphene nanoribbons [24] through the cellular membrane. However, we did not observe any significant leaking of LDH from cells or any increase in the number of dead cells, assessed by TBEA, which indicates that the cell membrane integrity in our experiment has not been disrupted. Similarly, no LDH leakage was observed, as described by Zhang et al. [15] after 24 h of exposure to graphene nanosheets, by Peruzynska et al. [11] after 48 h of exposure to one- and four-layer PEGylated graphene oxide (GO) nanoflakes, and by Chong et al. [25] after 24 h of exposure to graphene quantum dots. On the other hand, a significant release of LDH after exposure to GO was described by Zhang and Gurunathan [26]. They found about a 40% increase in LDH leaking caused by 24 h of exposure to 50 µg/mL of reduced GO. Gurunathan et al. [4] also reported a significant increase in LDH-leaking cells with a GO concentration of 20 µg/mL.

G-BNMs demonstrate a very wide variability of structures and chemical and physical properties. The cell lines used, different exposure conditions, surface residues and/or lipopolysaccharide contamination, used surfactants and the formation of protein biocorona also have significant roles in influencing their toxic effects. Due to the interaction of these factors, the results of G-BNMs toxicity studies are still inconsistent.

For example, Zhang et al. [15] observed the cytotoxic effect of a graphene nanosheet on neuronal PC12 cell lines, and Burgum et al. [27] found the cytotoxic effect of few-layer pristine graphene on activated THP-1 cells. Lin et al. [28] reported cytotoxic effects of few-layer graphene on primary macrophages, Lasocka et al. [29] reported the cytotoxicity of pristine graphene monolayer without sharp edges on the murine fibroblast L929 cell line, Demir and Marcos [30] reported the cytotoxicity of graphene nanoplatelets on the mouse lymphoma cell line and Malanagahalli et al. [31] reported the cytotoxicity of few-layer graphene on mouse macrophages. On the other hand, Zhang et al. [15] did not find any significant damage to the cell membrane, and Luo et al. [9] reported the absence of cytotoxicity of GO (smaller than 500 nm) regarding activated THP-1 cells.

Direct cell membrane damage (due to the sharp edges of GP), direct interaction between the hydrophobic surface of GP and cell membrane lipids [3], and the extraction of cholesterol molecules from the cell membrane by hydrophobic interaction between pure GP and the cholesterol tail [5] are all important mechanisms of GP cytotoxicity. All the above-mentioned mechanisms lead to membrane destabilization and a loss of cell integrity. We observed no potential of pristine GP, up to a concentration of 60 µg/mL, to disrupt cell membrane integrity.

In addition, as mentioned above, the cytotoxicity of GP can be strongly influenced by the formation of protein biocorona, which usually increases their biocompatibility [32]. In our experiments, we used a cultivation medium that contained 10% of fetal bovine serum. It is likely that bovine serum forms protein biocorona around the GP (change of average ζ-potential; Table 1) and in this way reduces their cytotoxicity.

### 4.2. Oxidative Stress

Another important mechanism of the GP toxic effect includes the generation of intracellular reactive oxygen species (ROS). The ROS are primed to destroy xenobiotics in cells; however, they can also damage protein structures and nucleic acids, triggering apoptotic and necrotic mechanisms and leading to cell death [2,3].

It is obvious that the level of the toxic effect of ROS is modified by the efficiency of antioxidant systems [3]. To assess the degree of induction of ROS (due to exposure to GP) we used as a model the level of glutathione (GSH), which is an important component of the antioxidant system. The total GSH reserves in cells are influenced by the inhibition of GSH biosynthesis, through an increased level of oxidation processes (ROS) and by inactivating the genes encoding the enzymes of GSH synthesis [33].

Data from the literature regarding the induction of oxidative stress (ROS) after exposure to G-BNMs are still inconsistent. Some works confirm this induction [4,15,34,35], while others do not [9,30]. In our experiments, we did not observe an “individual” decrease in free GSH in combination with an adequate increase in GSSG levels. This suggests that the reason for the decrease in total GSH and GSSG, as well as free GSH, after GP1 exposure (but not after GP2 exposure) was not the liquidation of ROS, but was probably an increased consumption of GSH during the reparation of damage to DNA (as found in the CBMN test). This presumption is supported by the absence of an inflammatory reaction (see Section 4.4, Inflammatory Potential), which is usually induced by oxidative stress (ROS) [36].

After exposure to GP2, we did not find any changes in total GSH, GSSG and free GSH levels, unlike exposure to GP1. We believe that this phenomenon was due to the different structure (and associated physicochemical properties) of GP2.

### 4.3. Cytostatic and Genotoxic Potential

#### 4.3.1. Cytostatic Potential

The CBMN test represents one of the most robust, fast, cheap, very sensitive and accurate techniques for testing cytotoxicity or cytostasis and genotoxicity assessment. There are nine biomarkers of cytotoxic and genotoxic potential that can be measured [37]. We are aware that the individual indexes are only another expression of the same fact, but we consider it appropriate to publish them all for comparison. According to the recommendation of Gonzalez et al. [38], to avoid a decrease in the cellular uptake of GP as a consequence of actin inhibition after the addition of cytochalasin B, we used a delayed co-treatment experiment protocol.

The presence of *BNC* and multinucleated cells confirmed that a duration of exposure of 30 h to cytochalasin B is enough to ensure the division of THP-1 cells (the mean/median proportion of *BNC* and multinucleated cells was about 74%/80% in the negative control; in the highest GP concentration it was about 60%). Therefore, we can assume that a duration of GP exposure of 40 h without cytochalasin B is appropriate to ensure contact between the GP and nuclear material or mitotic apparatus of THP-1 cells in our study, as recommended by Gonzalez et al. [38] and the OECD [18].

The *NDI* of nonexposed control THP-1 cells counted in our study (mean ± standard deviation/median 1.91 ± 0.05/1.92) was lower than the *NDI* of the control THP-1 cell group (1.99) counted by Senapati et al. [39]. We must keep in mind that the absolute value of *CBPI* and *NDI* depends on the time of cultivation with cytochalasin B; more specifically, on the number of cell divisions during the time of this cultivation.

The results of the LDH assay and TBEA are inconsistent with the results obtained by *CBPI*, *RI* and *NDI*, in that a non-significant decrease in the cell proliferation potential was observed. We can assume that the cell viability measured in our study by the membrane damage of cells did not correspond to cell proliferation and cytostasis. This problem was also pointed out by O’Donovan [40], Di Bucchianico et al. [41], and Ventura et al. [42]. The observation of non-corresponding cytotoxic and cytostatic (antiproliferation) potential may be caused by the more sensitive detection of changes in proliferation potential when using a visual assessment of *CBPI*, *RI* and *NDI*, or by the fact that the cells are alive, the cell membrane is not damaged, but the cells are less effective in proliferation and nuclear (cell) division (which could also be caused by the reparation of DNA damage), or that they will die after a longer time period. This assumption supports the observations of Wang et al. [43] and of Tian et al. [8]. In addition, the accumulation of exogenous materials may interrupt the normal cell division process [44]. The biodegradability of G-BNMs is still unclear. Some authors assume partial biodegradability during the inflammation reaction [1], while others suppose no biodegradability and persistence in an organism [44,45].

Similar results were noted regarding the dose-dependent reduction in *NDI* as reported by Heshmati et al. [46] after 48 h exposure of the HT29 cell line to GO nanosheets. About a 25% decrease in the proliferation activity of THP-1 cells was also described by Gurunathan et al. [4] after 24 h of exposure to GO. Burgum et al. [27], who obtained the opposite results, noted only a slight decrease in the percentage of cytostasis, based on *CBPI*.

The cytotoxicity of the maximal concentration of tested material during genotoxicity testing should not exceed 45 ± 5%, assessed by a reduction in *CBPI* or *RI* in the negative control [18]. In our experiment, the cytotoxicity assessed by *CBPI* and *RI* was maximally about 20%. We can assume that we used a concentration without significant cytotoxic potential. Burgum et al. [27] suggest that only a decrease in cell viability of under 80% of the control group will indicate a cytotoxic effect.

#### 4.3.2. Genotoxic Potential

The danger of interference with the CBMN test could present GP agglomerates or aggregates that can potentially mimic MN, but they are usually clearly distinguishable with an optical microscope. Therefore, automatic analysis is not recommended [21]. The background level of MN in a cell line suitable for CBMN, as used in genotoxicity testing, should not exceed 2% [27]. In the THP-1 cell line used in our study, the MN level was around 0.6% (maximal level was 0.9%). We can conclude that the THP-1 cell line is suitable for genotoxicity testing.

The basal level of the number of MN on 1000 *BNC* for THP-1 cells, as reported in the literature, is (mean ± standard deviation) 7.33 ± 0.33 [39]. In our study, the basal level was similar (6.00 ± 1.91).

We supposed that the lowest observed genotoxic effect level (LOGEL) for GP1 was above 5 µg/mL and, for GP2, 30 µg/mL, and that this genotoxic potential increases in a dose-dependent manner.

Similar results showing a dose-dependent increase in the number of *BNC* with MN, NBUDs and NPBs were described by Heshmati et al. [46] after 48 h of exposure of the HT29 cell line to GO nanosheets. A dose-dependent increase in the level of MN in *BNC* was also observed by Burgum et al. [27] after 24 h of exposure of the TT1 cell line to few-layer pristine graphene. The DNA damage was significant from 20 µg/mL [27].

Our results are consistent with the results of the study by Ursini et al. [47], who observed the occurrence of MN more often in a group of six workers who were occupationally exposed to graphene.

Akhavan et al. [24] assume that the genotoxic potential of graphene nanoribbons in human mesenchymal stem cells is caused by the penetration of sharp-edged graphene nanoribbons into the cells or into the nucleus. If the nanomaterial can reach and enter the nucleus, it could damage the DNA directly and cause genotoxicity [5]. We did not observe any GP in the nuclei of activated THP-1, and free GP2 were found only sporadically in the cytoplasm [14]. Because nonactivated TPH-1 cells are less effective in phagocytosis and the accumulation of GP in endosomes is lower, we can suppose the same localization of GP in this case. Both tested GPs were not able to penetrate directly through the nuclear membrane into nuclei in the interphase.

The information about DNA damage caused by pristine graphene platelets is very limited. We suggest that one possible mechanism is a direct interaction with naked nuclear DNA during cell division, or a direct mechanical influence on the cytoskeleton, leading to interaction with the mitotic apparatus of cells [8,44,48]. These interactions could cause an increase in DNA damage and lead to a dose-dependent increase in the number of MN in *BNC*. The dose-dependent increase in levels of NBUDs and NPBs supports the theory regarding the direct interaction of graphene with the cytoskeleton and mitotic apparatus.

Nonsignificant higher levels of *BNC* with MN, NBUDs and NPBs were noted for the smaller GP1 than for the bigger GP2. Our observation confirmed the finding of Akhavan et al. [49], who reported that smaller reduced GO platelets are more cytotoxic and genotoxic than bigger ones. They noted not only dose- but also size-dependent toxicity [49]. The lateral size of GP1 particles is 80–300 nm, and the thickness is 1–4 nm (lateral size of GP2 particles 250–400 nm, thickness also 1–4 nm), the content of oxygen is higher in GP1 (about 7.5% and, respectively, about 4.5% in GP2) and there are more defects in the structure of GP1 [14]. Therefore, we suppose that the genotoxicity of graphene platelets may not only be affected by dose and particle size but also by the content of contaminants and the presence of defects in the structure.

We also observed that a naked nucleus without cytoplasm is more often surrounded by clusters of GP (Figure 6d). It is questionable whether these clusters of GP are the reason for or as a consequence of cytoplasm loss.

The use of the CBMN assay for the detection of DNA damage and cytostasis induced by pristine graphene platelets is very rare in the literature. Only a few studies have been conducted [27,34].

According to our results, we suggest that CBMN may represent an effective, reproducible, sensitive, and low-cost method for primary screening for the cytotoxicity/cytostasis and the genotoxicity of nanomaterials. One of the biggest advantages of this method is the absence of demanding equipment.

There is a need to confirm these results with another assay detecting DNA damage and in vivo before introducing these two types of GP in practical applications where there is a risk of human exposure.

### 4.4. Inflammatory Potential

The verification of possible endotoxin contamination is an essential step in the evaluation of the immunomodulatory potential of NMs [1]. Due to their large adsorption capacity, NMs including GP may carry a large range of contaminants that affect the results of proinflammatory tests. This may lead to misleading conclusions about their pro-inflammatory potential [50,51]. It may also explain the frequent contradictory results obtained from testing similar NMs. Therefore, we verified the absence of LPS in both samples of GP, using a cell-based assay prior to all experiments. The verified concentration of LPS was determined to be below 0.1 EU/mL in all tested concentrations of both GP and should therefore induce no adverse reaction [52].

The absence of microbial contaminants was indirectly confirmed by an examination of supernatants collected from THP-1 that were exposed to GP. The cell-based assay and ELISA did not prove the release of proinflammatory cytokines IL-6 and TNFα (Figure 7a,c), respectively, which represent pyrogens that are typical in bacterial stimulation. Similarly, there was no release of IL-10 as an anti-inflammatory cytokine (Figure 7b). These results also suggest that there is either no or insignificant oxidative stress after treatment with GP. This corresponds with the findings of several studies pointing to a possible hemocompatibility of pristine GP, where, despite its intracellular persistence, there was no release of IL-6, TNF-α and IL-1β in either short-term or long-term exposures [31,53]. On the contrary, oxidized forms of graphene, like GO, have the capacity to induce a pro-inflammatory response via oxidative stress, a leading mechanism of cytotoxicity [35,54]. On the other hand, pristine graphene that lacks specific functional groups on its surface may interact non-specifically with organelles and cause disruption, depending on its shape and size [45,55]. This may correspond with our findings of increased DNA damage despite the absence of acute cytotoxicity and oxidative stress.

## 5. Conclusions

The aim of the presented article was to bring new cytotoxic, cytostatic, genotoxic and immunotoxic data concerning in vitro exposure of the human THP-1 cell line to two types of GP.

Cytotoxicity: after the exposure of THP-1 to GP, we did not find any significant leaking of LDH from cells or any increase in dead cells, as assessed by TBEA. This indicates that the cell membranes’ integrity has not been disrupted. We also found no evidence of the induction of oxidative stress due to GP exposure.

Cytostasis: we did not observe any significant increase in cytostasis (using the CBMN test). However, to confirm this conclusion, further experiments need to be performed on proliferating cells over a longer exposure period.

Genotoxicity: we found a significant dose-dependent increase in DNA damage (using the CBMN test). The lowest observed genotoxic effect level (LOGEL) for GP1 was above 5 µg/mL and, for GP2, above 30 µg/mL. DNA damage can be caused by the direct interaction of GP with naked DNA during cell division or by direct interaction with the cytoskeleton and mitotic apparatus.

Immunotoxicity: changes in the IL-6, IL-10 and TNF-α levels were not significant and indicated the absence of any induction of an immune response and/or the induction of oxidative stress. We did not detect any microbial contamination of GP samples that would induce an immune response (IL-6, IL-10, TNF-α).

## Figures and Tables

**Table 1:** Physical properties of GP [14].

Nanomaterial	Particle Size (nm) (Z-Average)	PdI	Average *ζ*-Potential (mV)	Average *ζ*-Potential (mV) (in Full RPMI)
GP1	178.5 ± 103	0.188	≤−50	≤−9
GP2	332 ± 85	0.293	≤−47	≤−11

## Data Availability

The data presented in this study are available upon request from the corresponding author.

## Supplementary Materials

The following are available online at https://www.mdpi.com/article/10.3390/nano11092210/s1, Figure S1: WST-1 activity of THP-1 after 40 h of incubation.
