# Supplementary material for: In Vitro Assessment of the Genotoxic Potential of Pristine Graphene Platelets
Source: Nanomaterials (Basel). 2021 Aug 27;11(9):2210. doi: 10.3390/nano11092210 (PMC8470272; doi:10.3390/nano11092210)
Supplement: Supplementary file 1 [file nanomaterials-11-02210-s001.zip › nanomaterials-1328729-SI.pdf]

nanomaterials

MDPI

Supplementary data

In Vitro Assessment of the Genotoxic Potential of Pristine Graphene Platelets

Andrea Malkova [1,2,*], Tereza Svadlakova [1,3], Avni Singh [3], Martina Kolackova [3], Radka Vankova [3], Pavel Borsky [1,2], Drahomira Holmannova [1], Adam Karas [1], Lenka Borska [1] and Zdenek Fiala [1]

[1] Institute of Preventive Medicine, Faculty of Medicine in Hradec Kralove, Charles University, 50003 Hradec Kralove, Czech Republic; svadlakovat@lfhk.cuni.cz (T.S.); BORSKYP@lfhk.cuni.cz (P.B.); holmd9ar@lfhk.cuni.cz (D.H.); karasad@lfhk.cuni.cz (A.K.); borka@lfhk.cuni.cz (L.B.); fiala@lfhk.cuni.cz (Z.F.)

[2] Institute of Pathological Physiology, Faculty of Medicine in Hradec Kralove, Charles University, 50003 Hradec Kralove, Czech Republic

[3] Institute of Clinical Immunology and Allergology, University Hospital Hradec Kralove and Faculty of Medicine in Hradec Kralove, Charles University, 50005 Hradec Kralove, Czech Republic; singhav@lfhk.cuni.cz (A.S.); kolackovam@lfhk.cuni.cz (M.K.); vankovr@lfhk.cuni.cz (R.V.)

* Correspondence: Malka8AR@lfhk.cuni.cz; Tel.: +42-(04)-95816373

1. Supplementary data

1.1. Cell Viability/Cytotoxicity (WST-1)

For evaluation of possible interferences, previously untreated cells were incubated with WST-1 reagent together with addition of GP for 3 h. Suspensions were further centrifuged for 10 000g for 10 min to get rid of GP and transferred in a new flat bottom 96-well plate. Absorbance was measured in a microplate spectrophotometer Synergy HTX (Biotek, USA) at 440 nm, with 690 nm set as the reference wavelength.

The data related to mitochondrial activity of THP-1 cells using WST-1 assay indicated decrease in cells metabolic activity as shown in Figure S1a and possible cytotoxic potential of both GPs over the exposure period of 40h. These results are inconsistent with the results of other cytotoxic tests. Therefore, we measured possible interferences. The results proved decrease of WST-1 activity in presence of GP1 but not in presence of GP2 (Figure S1b).

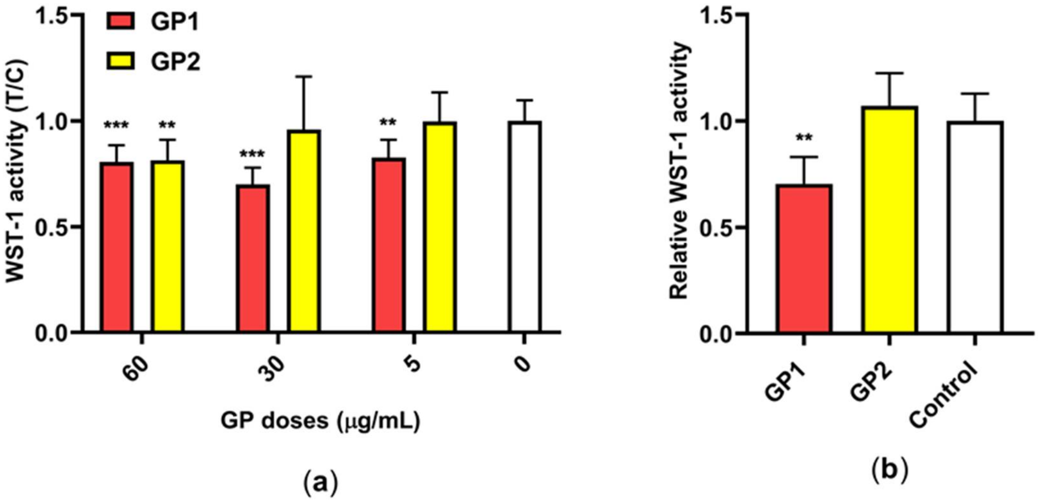

Figure S1. WST-1 activity of THP-1 after 40 h incubation. (**a**) THP-1 in response to GP (T; 5–60 µg/mL) after 40 h; (**b**) WST-1 activity of control THP-1 in presence of GP. Data are normalised to the corresponding controls and presented as mean ± standard deviation. ** p-value < 0.01; *** p-value < 0.001.
